# Supplementary figures and images for: Bacterial TonB-dependent transducers interact with the anti-σ factor in absence of the inducing signal protecting it from proteolysis
Source: PLoS Biol. 2024 Dec 2;22(12):e3002920. doi: 10.1371/journal.pbio.3002920 (PMC11637429; doi:10.1371/journal.pbio.3002920)

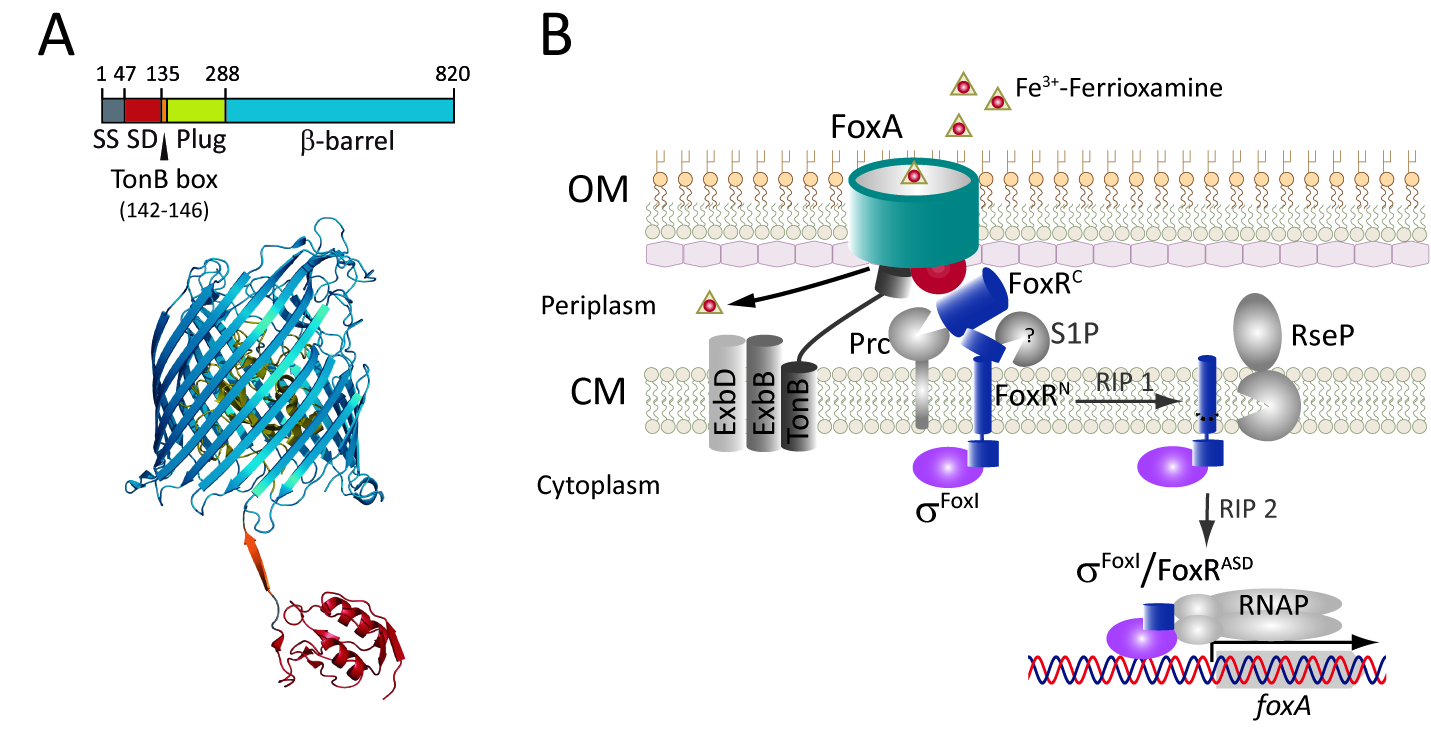

Supplement: S1 Fig — (A) Structure of the FoxA TBDT. The 22-stranded antiparallel β-barrel domain of FoxA is shown in blue and the plug domain occluding the pore in yellow. The signaling domain (SD) is shown in red and the TonB box in orange. Structure was solved in [10] and was downloaded from Protein Data Bank (PDB code 6I97). (B) Schematic representation of the Fox system. The 3 components of the CSS system—receptor (FoxA), anti-σ factor (FoxR), and σECF (σFoxI)—are shown as well as the TonB-ExbBD complex and the proteases involved in the proteolysis of FoxR. Upon synthesis, the P. aeruginosa anti-σ factor FoxR undergoes a spontaneous cleavage that produces 2 functional N- and C-domains (FoxRN and FoxRC) that interact with each other in the periplasm and are both required for proper function. The FoxA receptor interacts with the TonB protein, which enables the energy coupled uptake of the siderophore ferrioxamine, and with the FoxRC domain via its signaling domain (FoxASD, red ball). In response to ferrioxamine, FoxRC is degraded by the C-terminal periplasmic protease Prc, and this event triggers the regulated intramembrane proteolysis (RIP) of the FoxRN domain by the action of (at least) 2 proteases: a (still unidentified) site-1 protease (S1P) and the site-2 RseP protease (S2P). This results in the release of σFoxI into the cytoplasm bound to the anti-σ domain of FoxR (FoxRASD). Although not experimentally demonstrated yet, FoxRASD likely forms part of the transcription complex. Among other genes, σFoxI promotes the transcription of the foxA receptor gene. OM, outer membrane; CM, cytoplasmic membrane; RNAP, RNA polymerase. (TIF) [file pbio.3002920.s001.tif]

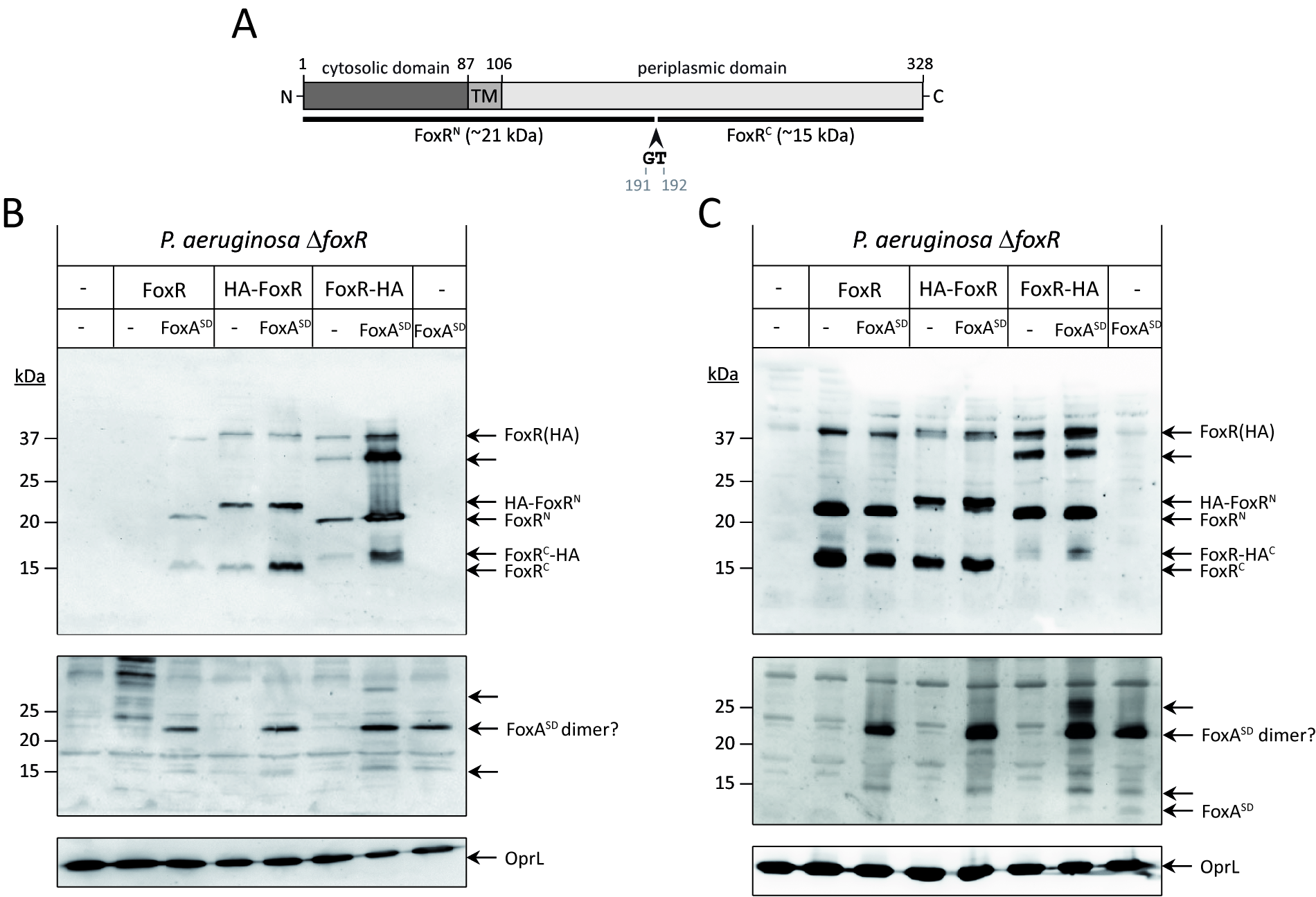

Supplement: S2 Fig — (A) Scheme of the P. aeruginosa FoxR protein. The cytosolic, transmembrane, and periplasmic regions of the protein are shown. The numbers indicate amino acid positions. The site at which the self-cleavage of FoxR occurs (between Gly-191 and Thr-192) is indicated. The N- and C-domains resulting from self-cleavage are illustrated. (B, C) Western blot analyses of the P. aeruginosa FoxR protein in cells overproducing FoxASD. The P. aeruginosa ΔfoxR mutant containing the pMMB67EH empty plasmid (-) or its derivate expressing FoxR, HA-FoxR, or FoxR-HA, and the pBBR1MCS-5 empty plasmid (-) or its derivative expressing FoxASD were grown in iron-restricted medium supplemented with 1 mM IPTG and 1 μm ferrioxamine B (B) or with 50 mM FeCl3 (C). Proteins were separated by SDS-PAGE and immunoblotted against FoxR using the FoxRperi polyclonal antibody (upper panel) and FoxA using the FoxASD polyclonal antibody (middle panel). Detection of the outer membrane lipoprotein OprL was used as loading control. Positions of the protein fragments and the molecular size marker (in kDa) are indicated. Presence of the HA-tag adds ∼1 kDa to the molar mass of the protein fragments. Blots are representatives of 3 biological replicates (N = 3). The raw data underlying the graphs shown in the figure can be found at Mendeley Data repository (Mendeley Data, V1, 10.17632/nxh4c8ymnn.2). Western blot can be found in S1 Raw Images. (TIF) [file pbio.3002920.s002.tif]

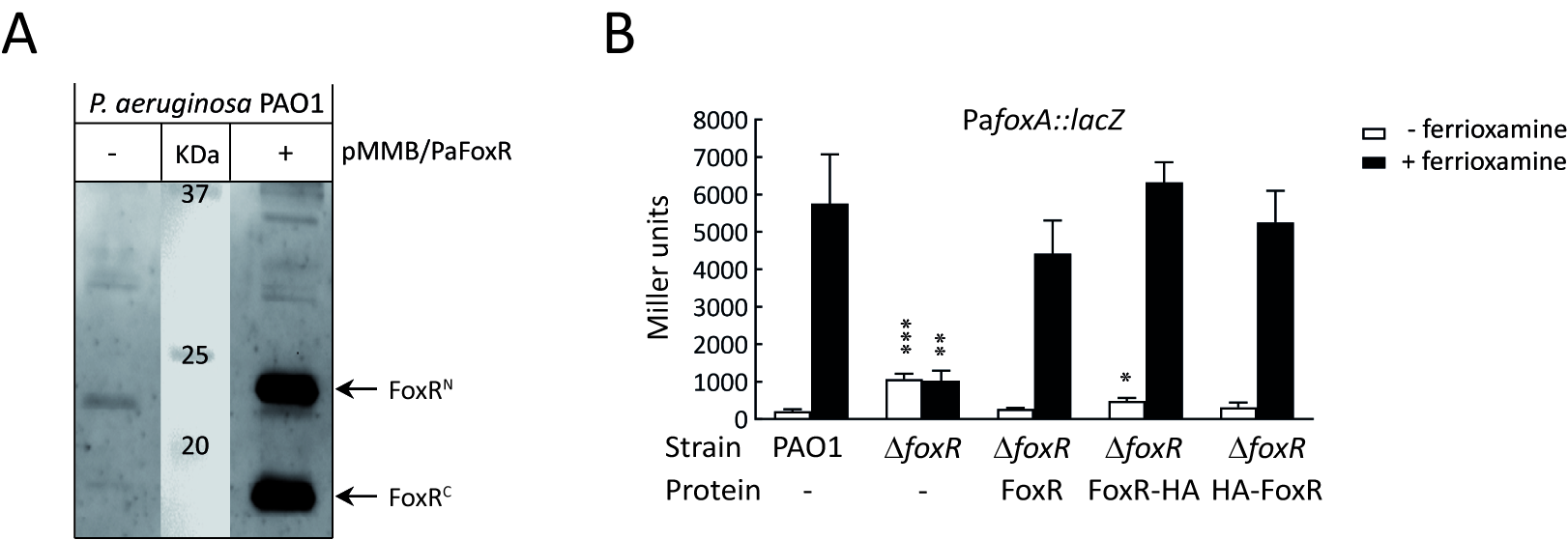

Supplement: S3 Fig — (A) P. aeruginosa PAO1 wild-type strain bearing the pMMB67EH empty plasmid or its derivative expressing the FoxR protein were grown under iron-restricted conditions and 1 mM IPTG. Proteins were immunoblotted against the anti-σ factor FoxR using a polyclonal antibody. Positions of the protein fragments and the molecular size marker (in kDa) are indicated. (B) The P. aeruginosa PAO1 wild-type strain and its isogenic ΔfoxR mutant bearing the foxA::lacZ transcriptional fusion and the pMMB67EH empty (-) or the pMMB67EH-derived plasmid producing the FoxR, FoxR-HA, or HA-FoxR proteins (S1 Table) were grown under iron-limitation conditions and in absence (-) or presence (+) of 1 μm ferrioxamine B. Activity was determined by β-galactosidase assay and data are means ± SD from 3 biological replicates (N = 3). P-values were calculated by two-tailed t test by comparing the value obtained in the PAO1 wild-type strain with that of the mutant strains in the same growth condition and are represented in the graphs by *, P < 0.05; **, P < 0.01; ***, P < 0.001; and ****, P < 0.0001. The raw data underlying the graphs shown in the figure can be found at Mendeley Data repository (Mendeley Data, V1, 10.17632/nxh4c8ymnn.2). Western blot can be found in S1 Raw Images. (TIF) [file pbio.3002920.s003.tif]

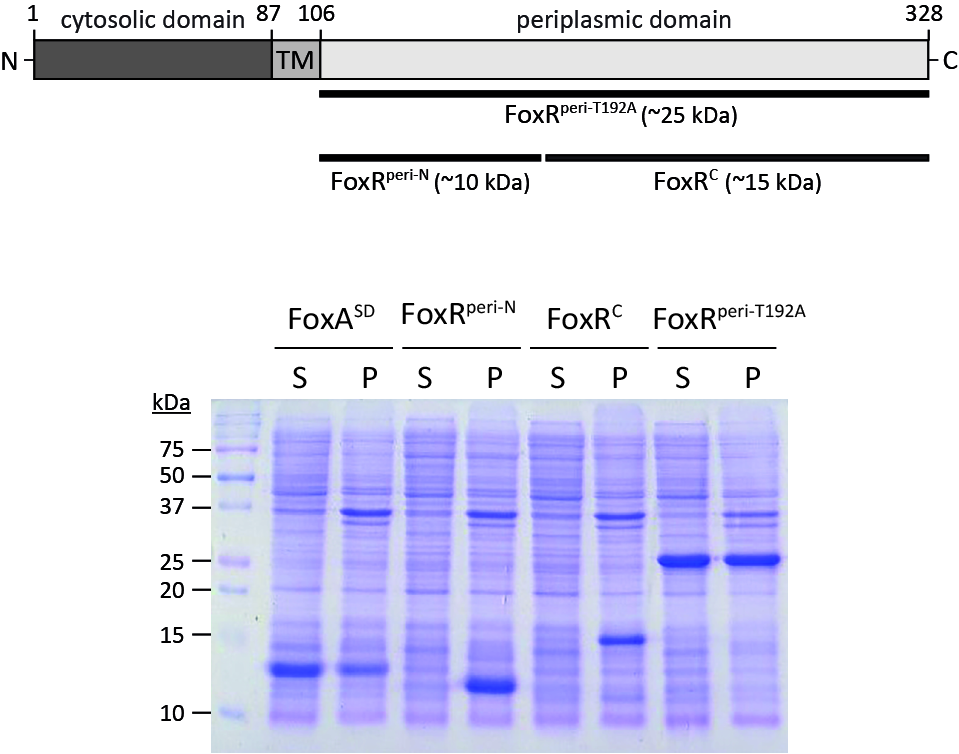

Supplement: S4 Fig — E. coli BL21 cells producing the indicated protein domain were grown as described in Materials and methods. Cultures were harvested, resuspended in buffer A at different pHs, and subjected to sonication. The gel shows protein samples from the soluble (S) and the pellet (P) fractions after cells were resuspended in buffer A at pH 8.0, while other trials with various pHs showed similar results. Position the molecular size marker (in kDa) is indicated. Western blot can be found in S1 Raw Images. (TIF) [file pbio.3002920.s004.tif]

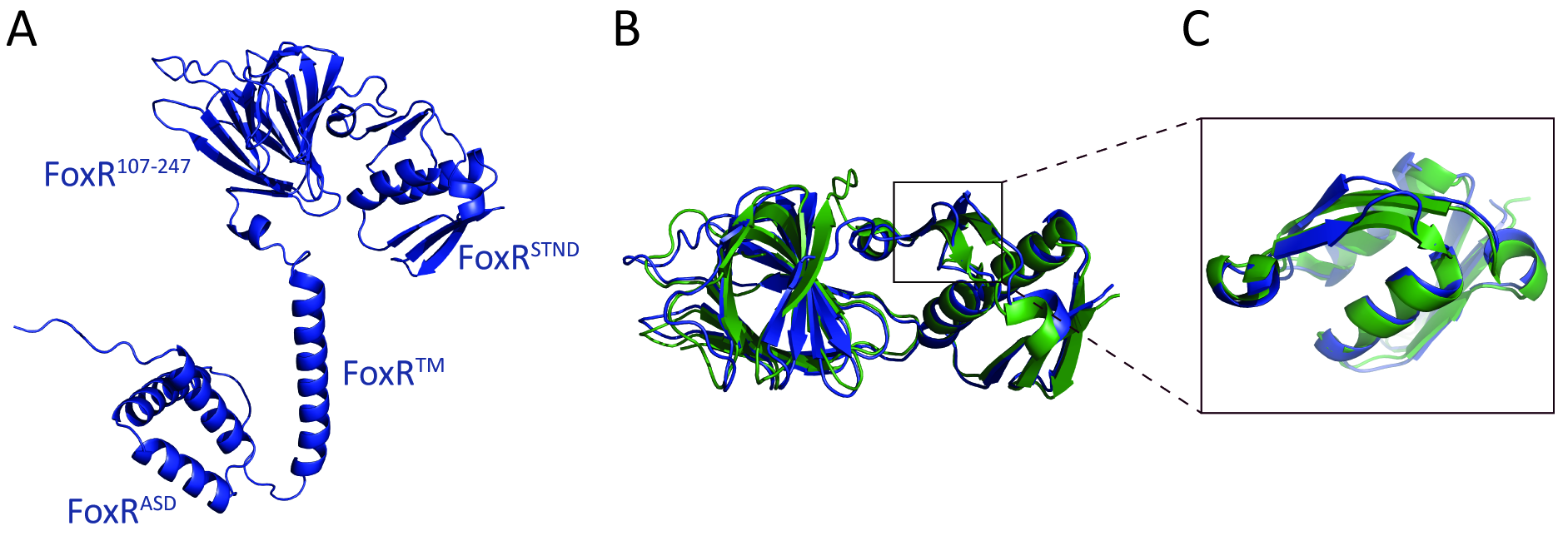

Supplement: S5 Fig — (A) The Alphafold protein structure of FoxR (AF_AFQ9I115F1) is shown. The different domains of the protein are indicated. (B, C) Structural alignment between the periplasmic portions of FoxR and PupR (6OVM) with focus on the 2 anti-parallel β strands of the STN domain (C). (TIF) [file pbio.3002920.s005.tif]

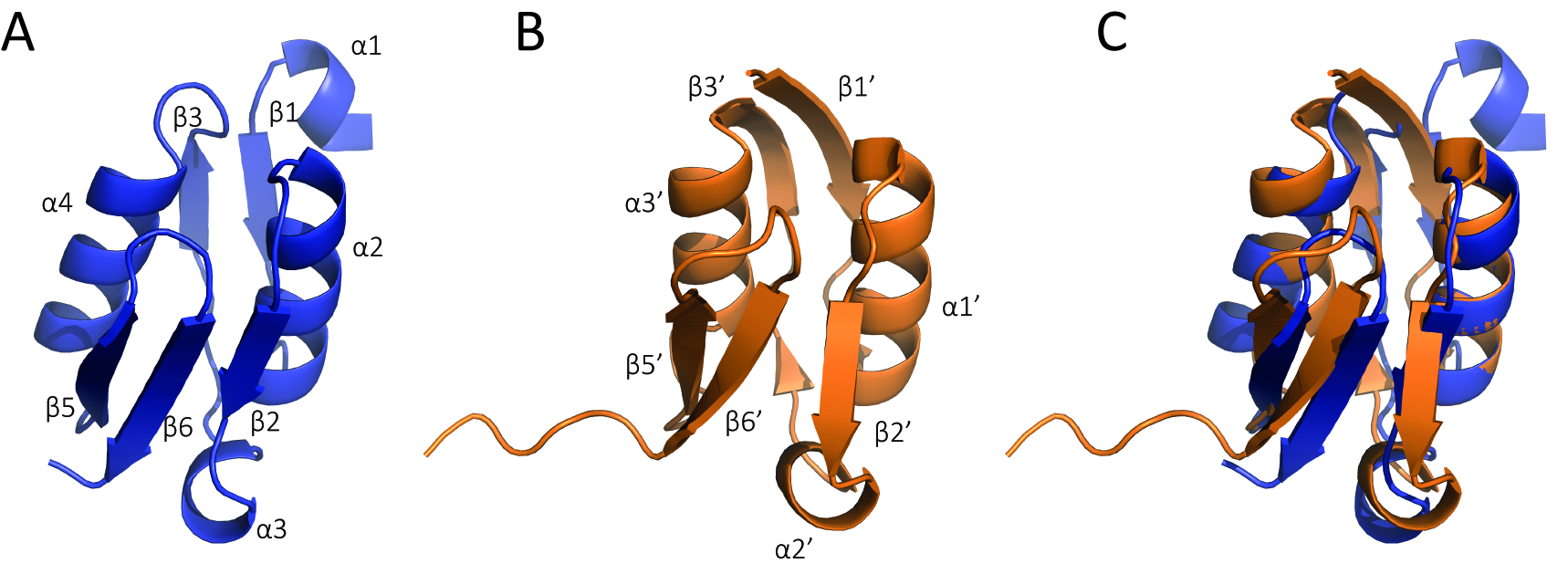

Supplement: S6 Fig — Structural features of the FoxRSTND (A) and FoxASD (B) protein structures (obtained from AF_AFQ9I115F1 and 6I97, respectively). (C) Structural alignment between both protein domains. (TIF) [file pbio.3002920.s006.tif]

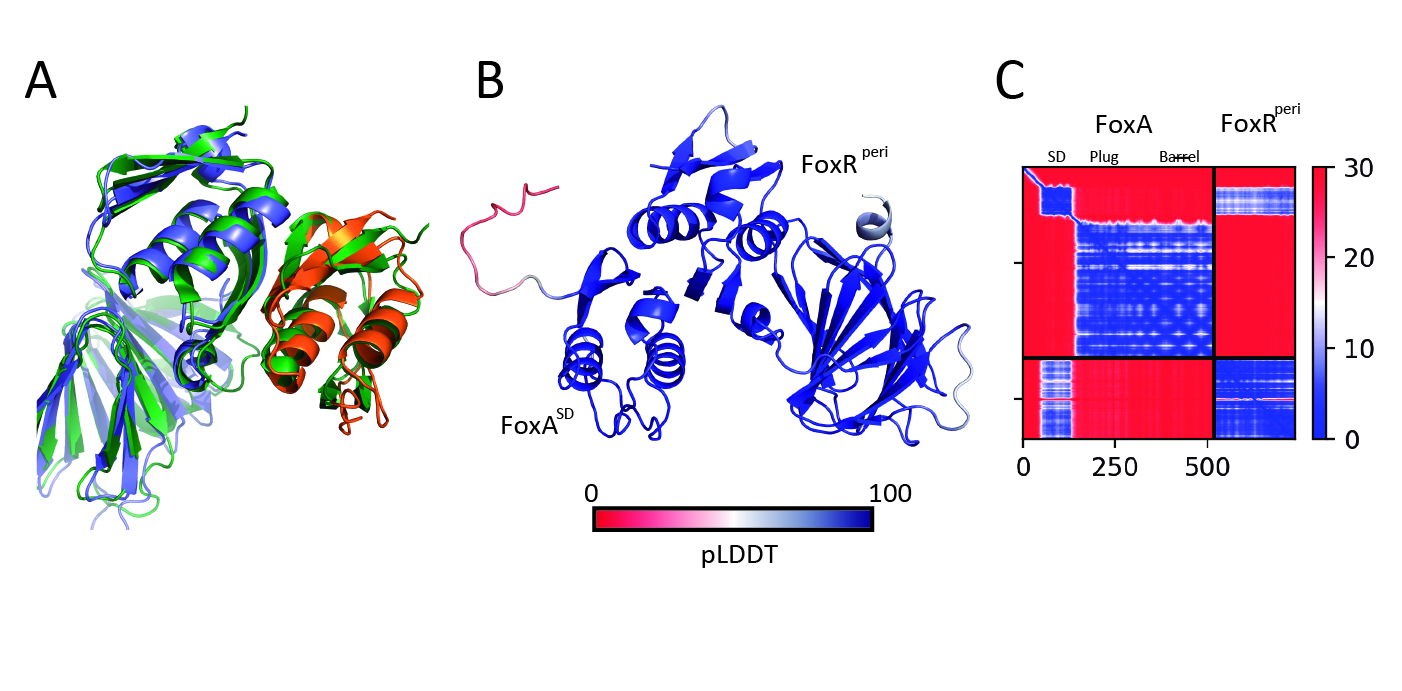

Supplement: S7 Fig — (A) Structural alignment between the Alphafold FoxRperi/FoxASD predicted complex (in blue and orange) and the PupB/PupR complex (in green, 6OVK) with an RSMD of 0.862. (B) Alphafold model for the FoxRperi/FoxASD complex colored by its pLDDT value. (C) PAE chart for the FoxRperi/FoxASD model showing a likely interaction between FoxASD and the FoxRperi proteins. The raw data underlying the graphs shown in the figure can be found at Mendeley Data repository (Mendeley Data, V1, 10.17632/nxh4c8ymnn.2). (TIF) [file pbio.3002920.s007.tif]

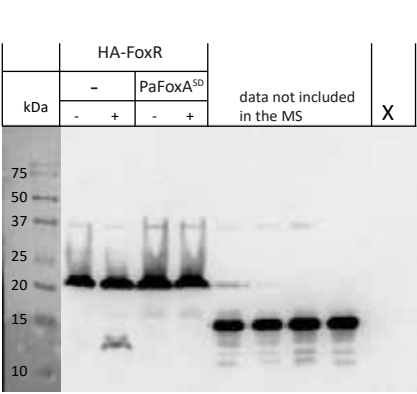

Fig. 1B

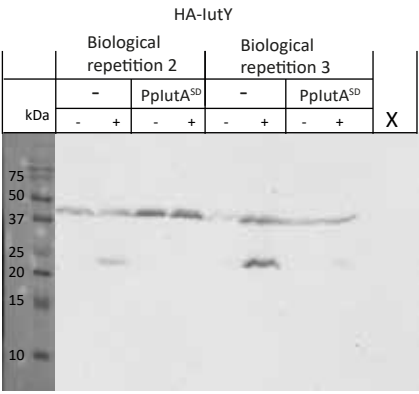

Fig. 1B

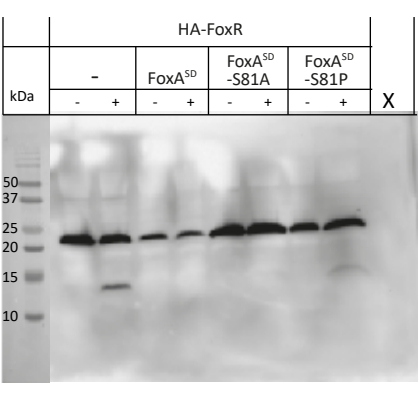

Fig. 4B

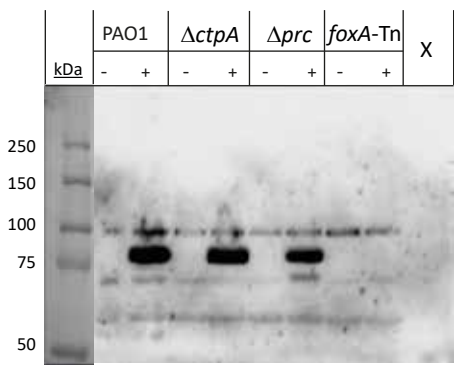

Fig. 4C

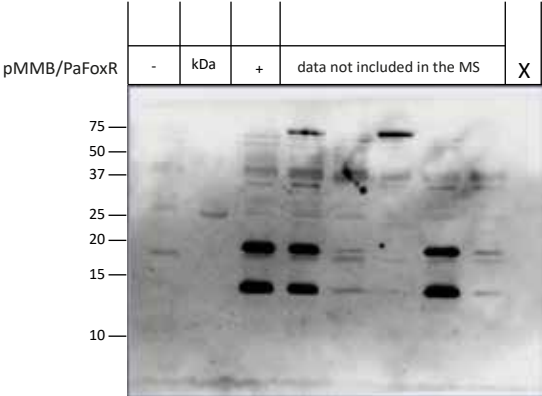

Fig. S3A

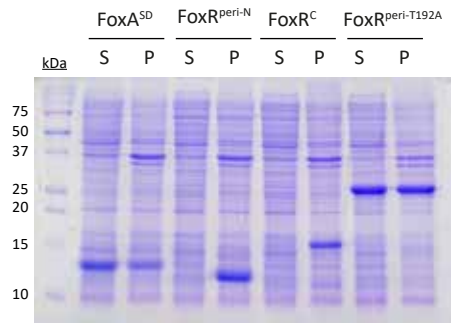

Fig. S4B

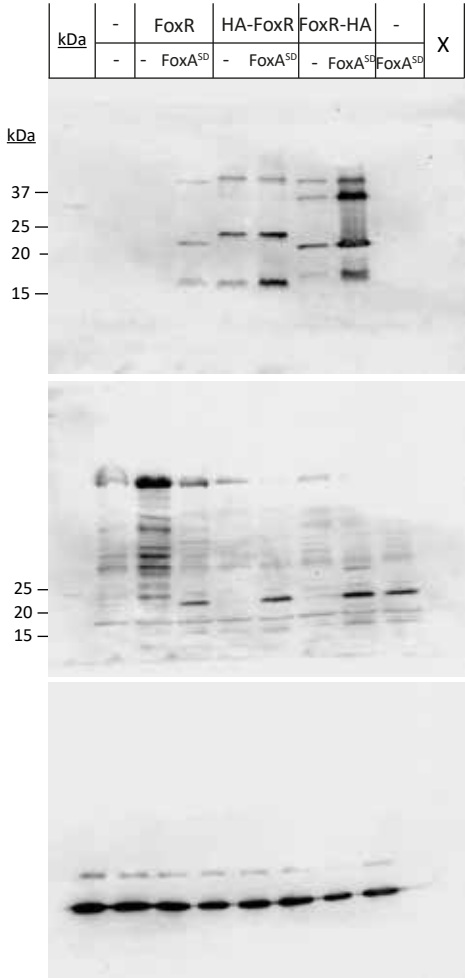

Fig. S2B

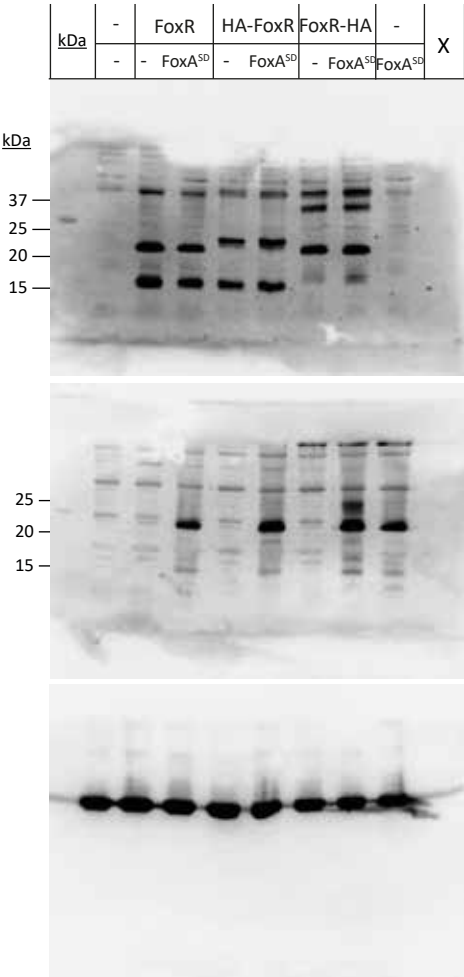

Fig. S2C

Supplement: S1 Raw Images — (PDF) [file pbio.3002920.s010.pdf]
