# Supplementary material for: Bacterial TonB-dependent transducers interact with the anti-σ factor in absence of the inducing signal protecting it from proteolysis
Source: PLoS Biol. 2024 Dec 2;22(12):e3002920. doi: 10.1371/journal.pbio.3002920 (PMC11637429; doi:10.1371/journal.pbio.3002920)
Supplement: S1 Table — (PDF) [file pbio.3002920.s008.pdf]

Table S1. Bacterial strains and plasmids used in this study[a]

**Strain**	**Characteristics**	**Reference**
Escherichia coli		
BL21 (DE3)	F^- *lon ompT hsdS* (r_B^- m_B^-) *gal dcm* λ(DE3)	[1]
BTH101	Adenylate cyclase-deficient (*cya*) strain (F^- *cya-99 araD139 galE1 galK16 rpsL1 hsdR2 mcrA1 mcrB1*); Sm^R	[2]
CC118λ*pir*	Δ(*ara-leu*) *araD* Δ*lacX74 galE galK phoA20 thi-1 rpsE rpoB argE recA1*, lysogenized with λ*pir*; Rif^R	[3]
DH5α	*supE44 Δ*(*lacZYA-argF*)*U169* ϕ80 *lacZ*ΔM15 *hsdR17* (r_K^- m_K^+) *recA1 endA1 gyrA96 thi1 relA1*; Nal^R	[4]
Pseudomonas aeruginosa		
PAO1	Wild-type strain	[5]
Δ*foxR*	PAO1 with a complete deletion of *foxR* (PA2467)	This study
Δ*prc*	PAO1 with a complete deletion of *prc* (PA3257)	[6]
Δ*ctpA*	PAO1 with a complete deletion of *ctpA* (PA5134)	[7]
foxA-Tn	PAO1 with a transposon insertion in *foxA* (PA2466)	[8]
Pseudomonas putida		
KT2440	*hsdR*1, wild-type strain; Rif^R	[9]
**Plasmids**		
pBBR1MCS-5	*ori*TRK2; Gm^R	[10]
pBBR/SDIutA	pBBR1MCS-5 carrying in EcoRI-XbaI a 0.6-Kb PCR fragment containing the signalling domain (SD) of the *P. putida* IutA (PP2193) CSS receptor (amino acids 1-134); Gm^R	This study
pBBR/SDPpFoxA	pBBR1MCS-5 carrying in EcoRI-XbaI a 0.6-Kb PCR fragment containing the signalling domain (SD) of the *P. putida* FoxA (PP0160) CSS receptor (amino acids 1-138); Gm^R	This study
pBBR/SDPpFiuA	pBBR1MCS-5 carrying in EcoRI-XbaI a 0.66-Kb PCR fragment containing the signalling domain (SD) of the *P. putida* FiuA (PP0350) CSS receptor (amino acids 1-118); Gm^R	This study
pBBR/SDPaFoxA	pBBR1MCS-5 carrying in EcoRI-XbaI a 0.55-Kb PCR fragment containing the signalling domain (SD) of the *P. aeruginosa* FoxA (PA2466) CSS receptor (amino acids 1-136); Gm^R	This study
pBBR/SDPaFiuA	pBBR1MCS-5 carrying in EcoRI-XbaI a 0.66-Kb PCR fragment containing the signalling domain (SD) of the *P. aeruginosa* FiuA (PA0470) CSS receptor (amino acids 1-108); Gm^R	This study
pBBR/SDHxuA	pBBR1MCS-5 carrying in EcoRI-XbaI a 0.57-Kb PCR fragment containing the signalling domain (SD) of the *P. aeruginosa* HxuA (PA1302) CSS receptor (amino acids 1-111); Gm^R	This study

pET28b(+)	Translation vector for cloning and expressing recombinant proteins in *E. coli*. Contains a 6xHis fusion tag; Km^R	Novagen
pET/SDPaFoxA	pET28b(+) carrying in NdeI-NheI a 0.27-Kb NdeI-XbaI PCR fragment containing the signalling domain of the *P. aeruginosa* FoxA receptor (amino acids 48-136) N-terminally fused to a 6xHis tag; Km^R	This study
pET/PaFoxRperi-T192A	pET28b(+) carrying in NdeI-HindIII a 0.69-Kb PCR fragment containing the entire periplasmic domain (amino acids 107-328) of the *P. aeruginosa* FoxR (PA2467) anti-σ factor (with a T192A mutation that avoids the spontaneous cleavage of this protein) N-terminally fused to a 6xHis tag; Km^R	This study
pET/PaFoxRperi-N	pET28b(+) carrying in NdeI-HindIII a 0.26-Kb PCR fragment containing the N-part of the periplasmic domain of the *P. aeruginosa* FoxR anti-σ factor (amino acids 107-191) N-terminally fused to a 6xHis tag; Km^R	This study
pET/PaFoxRC	pET28b(+) carrying in NdeI-HindIII a 0.44-Kb PCR fragment containing the C-domain of the periplasmic domain of the *P. aeruginosa* FoxR anti-σ factor (amino acids 192-328) N-terminally fused to a 6xHis tag; Km^R	This study
pKNG101	Gene replacement suicide vector, *ori*R6K, *oriT*RK2, *sacB;* Sm^R	[11]
pKNG/PaΔfoxR	pKNG101 suicide plasmid containing a 1.26-Kb SpeI-ApaI fragment from pTOPO/PaΔfoxR (to create the *P. aeruginosa* Δ*foxR* mutant); Sm^R	This study
pKNT25	BACTH cloning vector. For construction of chimeric proteins in which a polypeptide is fussed to the N-terminal end of the T25 fragment of *Bordetella pertussis* adenylate cyclase CyaA (amino acids 1-224); Km^R	[12]
pKNT25-zip	BACTH positive control plasmid. Encodes the chimeric protein T25::zip containing the leucine zipper region of the yeast protein GCN4; Km^R	[12]
pKNT25/SDPaFoxA	pKNT25 carrying in EcoRI-XbaI a 0.3-Kb PCR fragment) containing the signalling domain (SD) of the *P. aeruginosa* FoxA (PA2466) receptor (amino acids 48-136). Encodes the chimeric protein $FoxA^{SD}$-T25; Km^R	This study
pKNT25/$FoxA^{SD}$-S81A	pKNT25/SDPaFoxA in which the serine 81 residue has been changed to alanine. Encodes the chimeric protein $FoxA^{SD}$-S81A-T25; Km^R	This study
pKNT25/$FoxA^{SD}$-S81P	pKNT25/SDPaFoxA in which the serine 81 residue has been changed to proline. Encodes the chimeric protein $FoxA^{SD}$-S81P-T25; Km^R	This study
pMMB67EH	IncQ broad-host range plasmid, *lacI^q*; Ap^R	[13]
pMMBK1-HA	IncQ-based replicon plasmid containing a N-terminally HA-tagged *P. putida iutY* gene; Ap^R	[6]
pMMB/FoxR	IncQ-based replicon plasmid containing the *P. aeruginosa foxR* gene; Ap^R	[14]

pMMB/HA-FoxR	IncQ-based replicon plasmid containing a N-terminally HA-tagged *P. aeruginosa foxR* gene; Ap^R	[14]
pMMB/FoxR-HA	IncQ-based replicon plasmid containing a C-terminally HA-tagged *P. aeruginosa foxR* gene; Ap^R	[14]
pMPK4	IncP-based replicon plasmid containing a *P. putida iutA::lacZ* transcriptional fusion; Tc^R	[6]
pMP-PPfoxA	IncP-based replicon plasmid containing a *P. putida foxA::lacZ* transcriptional fusion; Tc^R	[6]
pMP-PPfiuA	IncP-based replicon plasmid containing a *P. putida fiuA::lacZ* transcriptional fusion; Tc^R	[6]
pMPR8b	IncP-based replicon plasmid containing a *P. aeruginosa foxA::lacZ* transcriptional fusion; Tc^R	[8]
pMPFiuA	IncP-based replicon plasmid containing a *P. aeruginosa fiuA::lacZ* transcriptional fusion; Tc^R	[8]
pMPhxuA	IncP-based replicon plasmid containing a *P. aeruginosa hxuA::lacZ* transcriptional fusion; Tc^R	[15]
pCR2.1-TOPO	TA PCR cloning vector; Km^R	Thermo Fisher Scientific
pTOPO/PaΔfoxR	pCR2.1-TOPO carrying a ~1.16-Kb PCR fragment containing the regions up- and downstream the *P. aeruginosa foxR* gene; Km^R	This study
pTOPO/PafoxRΔ192-328	pCR2.1-TOPO carrying a ~1.13-Kb PCR fragment containing the regions up- and downstream the *P. aeruginosa* $foxR^C$ domain gene; Km^R	This study
pTOPO/PafoxRΔ323-328	pCR2.1-TOPO carrying a ~0.93-Kb PCR fragment containing the regions up- and downstream the *P. aeruginosa* $foxR^{323\text{-}328}$ domain gene; Km^R	This study
pUT18C	BACTH cloning vector. For construction of chimeric proteins in which a polypeptide is fussed to the C-terminal end of the T18 fragment of *Bordetella pertussis* adenylate cyclase CyaA (amino acids 225-399); Ap^R	[12]
pUT18C-zip	BACTH positive control plasmid. Encodes the chimeric protein T18::zip containing the leucine zipper region of the yeast protein GCN4; Ap^R	[12]
pUT18C/PaFoxR1-191	pUTC18C carrying in EcoRI-XbaI a ~0.58-Kb PCR fragment containing the N-domain of the *P. aeruginosa* FoxR (PA2467) anti-σ factor (amino acids 1-191). Encodes the chimeric protein T18-$FoxR^N$; Ap^R	This study
pUT18C/PaFoxR107-191	pUTC18C carrying in EcoRI-XbaI a ~0.28-Kb PCR fragment containing the N-part of the periplasmic domain of the *P. aeruginosa* FoxR (PA2467) anti-σ factor (amino acids 107-191). Encodes the chimeric protein T18-$FoxR^{peri\text{-}N}$; Ap^R	This study
pUT18C/PaFoxR192-328	pUTC18C carrying in EcoRI-XbaI a ~0.42-Kb PCR fragment containing the C-part of the periplasmic domain of the *P. aeruginosa* FoxR (PA2467) anti-σ factor (amino acids 192-328). Encodes the chimeric protein T18-$FoxR^C$; Ap^R	This study

pUT18C/PaFoxRperi-T192A	pUTC18C carrying in EcoRI-XbaI a ~0.69-Kb PCR fragment containing the periplasmic domain of the *P. aeruginosa* FoxR (PA2467) anti-σ factor (amino acids 107-328) with a T192A mutation that avoids the spontaneous cleavage of this protein. Encodes the chimeric protein T18-FoxR$^{peri-T192A}$; Ap^R	This study
pUT18C/PaFoxR192-255	pUTC18C carrying in EcoRI-XbaI a ~0.19-Kb PCR fragment containing the C-part of the periplasmic domain of the *P. aeruginosa* FoxR (PA2467) anti-σ factor (amino acids 192-255). Encodes the chimeric protein T18-FoxR$^{192-255}$; Ap^R	This study
pUT18C/PaFoxR256-328	pUTC18C carrying in EcoRI-XbaI a ~0.22-Kb PCR fragment containing the STN domain of the *P. aeruginosa* FoxR (PA2467) anti-σ factor (amino acids 256-328). Encodes the chimeric protein T18-FoxR^{STND}; Ap^R	This study
pUT18C/FoxR^C-S292A	pUT18C/PaFoxR192-328 in which the serine 292 residue has been changed to alanine. Encodes the chimeric protein T18-FoxR^C-S292A; Ap^R	This study
pUT18C/FoxR^C-S292P	pUT18C/PaFoxR192-328 in which the serine 292 residue has been changed to proline. Encodes the chimeric protein T18-FoxR^C-S292P; Ap^R	This study
pUT18C/FoxR^C-G293A	pUT18C/PaFoxR192-328 in which the glycine 293 residue has been changed to alanine. Encodes the chimeric protein T18-FoxR^C-G293A; Ap^R	This study
pUT18C/FoxR^C-G293P	pUT18C/PaFoxR192-328 in which the glycine 293 residue has been changed to proline. Encodes the chimeric protein T18-FoxR^C-G293P; Ap^R	This study

[a] Ap^R, Gm^R, Km^R, Nal^R, Rif^R, Sm^R and Tc^R, resistance to ampicillin, gentamycin, kanamycin, nalidixic acid, rifampicin, streptomycin, and tetracycline, respectively.

REFERENCES

1. Jeong H, Barbe V, Lee CH, Vallenet D, Yu DS, Choi SH, et al. Genome sequences of *Escherichia coli* B strains REL606 and BL21(DE3). J Mol Biol. 2009;394(4):644-52. Epub 2009/09/30. doi: 10.1016/j.jmb.2009.09.052. PubMed PMID: 19786035.

2. Ouellette SP, Karimova G, Davi M, Ladant D. Analysis of membrane protein interactions with a bacterial adenylate cyclase-based two-hybrid (BACTH) technique. Curr Protoc Mol Biol. 2017;118:20 12 1-20 12 24. doi: 10.1002/cpmb.36. PubMed PMID: 28369675.

3. Herrero M, de Lorenzo V, Timmis KN. Transposon vectors containing non-antibiotic resistance selection markers for cloning and stable chromosomal insertion of foreign genes in gram-negative bacteria. J Bacteriol. 1990;172(11):6557-67. PubMed PMID: 2172216.

4. Hanahan D. Studies on transformation of *Escherichia coli* with plasmids. J Mol Biol. 1983;166(4):557-80. PubMed PMID: 61.

5. Jacobs MA, Alwood A, Thaipisuttikul I, Spencer D, Haugen E, Ernst S, et al. Comprehensive transposon mutant library of *Pseudomonas aeruginosa*. Proc Natl Acad Sci U S A. 2003;100(24):14339-44. PubMed PMID: 64.

6. Bastiaansen KC, Ibañez A, Ramos JL, Bitter W, Llamas MA. The Prc and RseP proteases control bacterial cell-surface signalling activity. Environ Microbiol. 2014;16(8):2433-43. Epub 2014/01/01. doi: 10.1111/1462-2920.12371. PubMed PMID: 24373018.

7. Otero-Asman JR, Sánchez-Jiménez A, Bastiaansen KC, Wettstadt S, Civantos C, García-Puente A, et al. The Prc and CtpA proteases modulate cell-surface signaling activity and virulence in *Pseudomonas aeruginosa*. iScience. 2023;26(7):107216. doi: 10.1016/j.isci.2023.107216.

8. Llamas MA, Sparrius M, Kloet R, Jimenez CR, Vandenbroucke-Grauls C, Bitter W. The heterologous siderophores ferrioxamine B and ferrichrome activate signaling pathways in *Pseudomonas aeruginosa*. J Bacteriol. 2006;188(5):1882-91. PubMed PMID: 2.

9. Franklin FC, Bagdasarian M, Bagdasarian MM, Timmis KN. Molecular and functional analysis of the TOL plasmid pWWO from *Pseudomonas putida* and cloning of genes for the entire regulated aromatic ring meta cleavage pathway. Proc Natl Acad Sci U S A. 1981;78(12):7458-62. doi: 10.1073/pnas.78.12.7458. PubMed PMID: 6950388; PubMed Central PMCID: PMCPMC349287.

10. Kovach ME, Elzer PH, Hill DS, Robertson GT, Farris MA, Roop RM, et al. Four new derivatives of the broad-host-range cloning vector pBBR1MCS, carrying different antibiotic-resistance cassettes. Gene. 1995;166(1):175-6. PubMed PMID: 214.

11. Kaniga K, Delor I, Cornelis GR. A wide-host-range suicide vector for improving reverse genetics in gram-negative bacteria: inactivation of the *blaA* gene of *Yersinia enterocolitica*. Gene. 1991;109(1):137-41. doi: 10.1016/0378-1119(91)90599-7. PubMed PMID: 1756974.

12. Karimova G, Pidoux J, Ullmann A, Ladant D. A bacterial two-hybrid system based on a reconstituted signal transduction pathway. Proc Natl Acad Sci U S A. 1998;95(10):5752-6. PubMed PMID: 9576956; PubMed Central PMCID: PMCPMC20451.

13. Fürste JP, Pansegrau W, Frank R, Blocker H, Scholz P, Bagdasarian M, et al. Molecular cloning of the plasmid RP4 primase region in a multi-host-range *tacP* expression vector. Gene. 1986;48(1):119-31. doi: 10.1016/0378-1119(86)90358-6. PubMed PMID: 3549457.

14. Bastiaansen KC, Otero-Asman JR, Luirink J, Bitter W, Llamas MA. Processing of cell-surface signalling anti-sigma factors prior to signal recognition is a conserved autoproteolytic mechanism that produces two functional domains. Environ Microbiol. 2015;17(9):3263-77. Epub 2015/01/13. doi: 10.1111/1462-2920.12776. PubMed PMID: 25581349.

15. Otero-Asman JR, Garcia-Garcia AI, Civantos C, Quesada JM, Llamas MA. *Pseudomonas aeruginosa* possesses three distinct systems for sensing and using the host molecule haem. Environ Microbiol. 2019;21(12):4629-47. doi: 10.1111/1462-2920.14773. PubMed PMID: 31390127.
