# Supplementary material for: Bacterial TonB-dependent transducers interact with the anti-σ factor in absence of the inducing signal protecting it from proteolysis
Source: PLoS Biol. 2024 Dec 2;22(12):e3002920. doi: 10.1371/journal.pbio.3002920 (PMC11637429; doi:10.1371/journal.pbio.3002920)
Supplement: S2 Table — (PDF) [file pbio.3002920.s009.pdf]

Table S2. Sequences of the primers used in this study

Plasmid	DNA template[a]	Primer	Sequence (5' → 3')[b]
pBBR/SDIutA	KT2440 genomic DNA	PRPP_2193F	TTA**GAATTC**ATTGATCAGCCTGTTCCG
		SDIutAR-H	ACT**AAGCT**_TCA_CTGCGGCGCGGCCTCGG
pBBR/SDPpFoxA	KT2440 genomic DNA	Pr-PP0160F-E	AAT**GAATTC**TGCTACCGCAGACGTTGCC
		Pp-SDFoxAR-X	ACT**TCTAGA**_TCA_CAATGCCAGGGCGGCGTTC
pBBR/SDPpFiuA	KT2440 genomic DNA	Pr-PP0350F-E	AAA**GAATTC**GGCATACGCAGTGGTGGG
		Pa-SDFiuAR-X	ACT**TCTAGA**_TCA_CAAGGCCAGTGTGCCATCCG
pBBR/SDPaFoxA	PAO1 genomic DNA	PR85E2	TTT**GAATTC**CGCTTGCTTTCGTCCG
		Pa-SDFoxAR-X	ACT**TCTAGA**_TCA_CACATCGCCGTCCTTTGCC
pBBR/SDPaFiuA	PAO1 genomic DNA	PFiuA5E	AAT**GAATTC**TGGTGGTTCGAAGTGGTGCC
		Pa-SDFiuAR-X	ACT**TCTAGA**_TCA_CACCTTGCGCAGGCTGTAGG
pBBR/SDHxuA	PAO1 genomic DNA	PrPA1302F-E	AAA**GAATTC**CCCGACGATTTTCTCCTCGC
		SDHxuAR-X	ACT**TCTAGA**_TCA_GCTGTAGGCGTCGACCTGGC
pET/SDPaFoxA	pBBR/SDPaFoxA (Table S1, this study)	PaFoxA(-ss)F-Nd	AAA**CATATG**GCCGAAGCGGCGGCGGAACA
		pBBR1-R	GGTAACGCCAGGGTTTTCCC
pET/PaFoxRperi-T192A	pMMB/HA-FoxR-T192A [1]	PaFoxRperiF-Nd	AAA**CATATG**GATAGCCTGCCCTGGCAGCG
		MMB673'	TGTTTTATCAGACCGCTTC
pET/PaFoxRperi-N	pMMB/FoxRperi-CHA [1]	PaFoxRperiF-Nd	AAA**CATATG**GATAGCCTGCCCTGGCAGCG
		PaFoxRperiN-R-H	AAA**AAGCT**_TCA_GCCGAGGGCCTCGAACAGC
pET/PaFoxRC	pMMB/FoxRperi-NHA [1]	PaFoxRC-F-Nd	ACA**CATATG**ACCCGTTTCAACGTACGCC
		MMB673'	TGTTTTATCAGACCGCTTC
pTOPO/PaΔfoxR	PAO1 genomic DNA	dfoxR fw up (SWO11)	ATCATTCATGTGTCGCCCCG
		dfoxR ovlp rev (SWO14)	GACCACCCTACCGCGCGTC
	PAO1 genomic DNA	dfoxR ovlp fw (SWO13)	TAGGGTGGTCGCCGCCTG
		rev dn dfoxRC (SW04)	CGCCTGCATGTTCAGCACAT
pKNT25/SDPaFoxA	pBBR/SDPaFoxA (Table S1, this study)	SDPaFoxA(-ss)F-X	AAA**TCTAGA**_G_GCCGAAGCGGCGGCGGAACA
		SDPaFoxAR-E	AAA**GAATTC**_GA_CACATCGCCGTCCTTTGCCG
pKNT25/FoxA[SD]-S81A	pKNT25/SDPaFoxA (Table S1, this study	SDPaFoxA(-ss)F-X	AAA**TCTAGA**_G_GCCGAAGCGGCGGCGGAACA
		SDPaFoxA-S81A-R	CGGAGTGGCCG**C**GAGGGTGAT
	pKNT25/SDPaFoxA (Table S1, this study	SDPaFoxA-S81A-F	ATCACCCTC**G**CGGCCACTCCG
		SDPaFoxAR-E	AAA**GAATTC**_GA_CACATCGCCGTCCTTTGCCG
pKNT25/FoxA[SD]-S81P	pKNT25/SDPaFoxA (Table S1, this study	SDPaFoxA(-ss)F-X	AAA**TCTAGA**_G_GCCGAAGCGGCGGCGGAACA
		SDPaFoxA-S81P-R	CGGAGTGGCCG**G**GAGGGTGAT
	pKNT25/SDPaFoxA (Table S1, this study	SDPaFoxA-S81P-F	ATCACCCTC**C**CGGCCACTCCG
		SDPaFoxAR-E	AAA**GAATTC**_GA_CACATCGCCGTCCTTTGCCG
pUT18C/PaFoxR1-191	PAO1 genomic DNA	PaFoxRF-X	AAA**TCTAGA**_G_GTGGACGGGACGCGCGGTAGGG
		PaNFoxR-R-E	AAA**GAATTC**_CGC_GCCGAGGGCCTCGAACAGC
pUT18C/PaFoxR107-191	PAO1 genomic DNA	PaFoxRperiF(-start)-X	AAA**TCTAGA**_G_GATAGCCTGCCCTGGCAGCG
pUT18C/PaFoxR192-328	PAO1 genomic DNA	PaFoxRC-F-X	AAA**TCTAGA**_G_ACCCGTTTCAACGTACGCC
		PaFoxRC(-stop)-R-E	AAA**GAATTC**_GA_GGCGGCGACCACCCTCACCC

pUT18C/PaFoxRperi-T192A	pMMB/HA-FoxR-T192A [1]	PaFoxRperiF(-start)-X	AAA**TCTAGA**GGATAGCCTGCCCTGGCAGCG
		PaFoxRC(-stop)-R-E	AAA**GAATTC***GA*GGCGGCGACCACCCTCACCC
pUT18C/PaFoxR192-255	PAO1 genomic DNA	PaFoxRC-F-X	AAA**TCTAGA***G*ACCCGTTTCAACGTACGCC
		PaFoxR 255 R B	AAA**GGATCC***GA*ATCGGCCCAGGCCACGGC
pUT18C/PaFoxR256-328	PAO1 genomic DNA	PaFoxR256-328-F-X	AAA**TCTAGA***G*GGCCTCATCGTCACCCGC
		PaFoxRC(-stop)-R-E	AAA**GAATTC***GA*GGCGGCGACCACCCTCACCC
pUT18C/FoxR^{C}-S292A	pUT18C/PaFoxR 192-328 (Table S1, this study)	PaFoxRC-F-X	AAA**TCTAGA***G*ACCCGTTTCAACGTACGCC
		FoxR-S292A-Rn	GGCGATAGACCCCGG**C**CAGG
	pUT18C/PaFoxR 192-328 (Table S1, this study)	FoxR-S292A-Fn	CCTG**G**CCGGGGTCTATCGCC
		PaFoxRC(-stop)-R-E	AAA**GAATTC***GA*GGCGGCGACCACCCTCACCC
pUT18C/FoxR^{C}-S292P	pUT18C/PaFoxR 192-328 (Table S1, this study)	PaFoxRC-F-X	AAA**TCTAGA***G*ACCCGTTTCAACGTACGCC
		FoxR-S292P-Rn	GGCGATAGACCCC**G**G**G**CAGG
	pUT18C/PaFoxR 192-328 (Table S1, this study)	FoxR-S292P-Fn	CCTG**C**C**G**GGGGTCTATCGCC
		PaFoxRC(-stop)-R-E	AAA**GAATTC***GA*GGCGGCGACCACCCTCACCC
pUT18C/FoxR^{C}-G293A	pUT18C/PaFoxR 192-328 (Table S1, this study)	PaFoxRC-F-X	AAA**TCTAGA***G*ACCCGTTTCAACGTACGCC
		PaFoxR-G293A-R	CCAGGCGATAGAC**GG**CGGAC
	pUT18C/PaFoxR 192-328 (Table S1, this study)	PaFoxR-G293A-F	GTCCG**CC**GTCTATCGCCTGG
		PaFoxRC(-stop)-R-E	AAA**GAATTC***GA*GGCGGCGACCACCCTCACCC
pUT18C/FoxR^{C}-G293P	pUT18C/PaFoxR 192-328 (Table S1, this study)	PaFoxRC-F-X	AAA**TCTAGA***G*ACCCGTTTCAACGTACGCC
		PaFoxR-G293P-R	CCAGGCGATAGACC**GG**GGAC
	pUT18C/PaFoxR 192-328 (Table S1, this study)	PaFoxR-G293P-F	GTCC**CC**GGTCTATCGCCTGG
		PaFoxRC(-stop)-R-E	AAA**GAATTC***GA*GGCGGCGACCACCCTCACCC

[a] The reference is indicated

[b] The sequences of the restriction sites are indicated in bold

REFERENCES

1. Bastiaansen KC, Otero-Asman JR, Luirink J, Bitter W, Llamas MA. Processing of cell-surface signalling anti-sigma factors prior to signal recognition is a conserved autoproteolytic mechanism that produces two functional domains. Environ Microbiol. 2015;17(9):3263-77. Epub 2015/01/13. doi: 10.1111/1462-2920.12776. PubMed PMID: 25581349.
