# Supplementary material for: Bacterial TonB-dependent transducers interact with the anti-σ factor in absence of the inducing signal protecting it from proteolysis
Source: PLoS Biol. 2024 Dec 2;22(12):e3002920. doi: 10.1371/journal.pbio.3002920 (PMC11637429; doi:10.1371/journal.pbio.3002920)
Supplement: Attachment — Submitted filename: Response to Reviewers_MLL_FJM_AO.docx [file pbio.3002920.s011.docx]

**Reviewer #1:**

The acquisition of iron by the uptake of siderophores in Gram-negative bacteria implies a Cell-Surface Signaling (CSS) process that transfers the extracellular environmental signal (the presence of the iron-siderophore) to the cytoplasm via a number of proteins localized in the outer membrane, the periplasm and the inner membrane, finally resulting in the release of a sigma factor that, in the absence of the signal, remains trapped by a membrane-bound anti-sigma factor. This sigma factor drives the RNA polymerase to a number of specific genes. The molecular details of ferric siderophore-mediated activation of the iron import machinery by the CSS process are complex and not fully clear. Using biochemical and gene expression (two-hybrids) approaches, and supported by structural models, in this manuscript authors provide experimental evidence that allows proposing some changes in the view on how the signal transducing process occurs. Previously, the model implied that the presence of the signal allows the interaction between the siderophore receptor located in the outer membrane (TBDT) and the anti-σ factor in the periplasm, promoting the proteolysis of the anti-sigma factor and in turn releasing the sigma factor. The results presented here allow proposing a somewhat different way of signal transduction in which sigma factor release does not depend on this interaction. Rather, the contact between the TBDT and the anti-σ factor would already occur in the absence of the signal (the iron-siderophore). This interaction would protect the anti-sigma from proteolysis by periplasmic proteases. To exert this role, the anti-sigma undergoes a self-catalysed proteolytic cleavage. Signal transduction involves further cleavage of the anti-sigma, probably by the CtpA protease. Initial evidence is provided for three different siderophore receptors in two different Pseudomonas species, and the molecular mechanisms are later worked out in more detail for one of them (the P. aeruginosa ferrioxamine FoxA receptor/transducer and the FoxR anti-sigma), defining the interacting domains.

In my view, the experiments and the results are clear, rigorous and convincing, and provide useful information. The manuscript is clearly written and is easy to follow in spite of the complexity of the problem.

**We thank the reviewer for this comment.**

Specific comments

1. Figure 1 was missing in the "R1" version of the manuscript that I received. Fortunately, the original "R0" version was also available to me at the PLoS web, and shows this figure, which is essential to follow the manuscript.

**Figure 1 is indeed in the R1 version but for an unknown reason it was loaded as the last figure instead of the first one. We did not note this when submitting the R1 version and we apologize for this inconvenience. Because the manuscript is presented as a short report following the editor’s recommendation, this figure has now been placed in supplementary information. We also consider it is an important figure to follow the manuscript.**

2. Page 7, line 144 onwards. Authors overproduce the signalling domain of the siderophore receptor and detect a resulting phenotype (inhibition of the CSS activity). I assume that this overproduction is done in a way that allows the signalling domain to be exported to the periplasm, but this is not specifically explained in the text. While this is probably obvious for the authors, it is not so for the readers, and it is important to know it to follow the line of reasoning. Please explain.

**We thank the reviewer for this important comment. Because all the constructs we made to perform this experiment contain the signal sequence, it was obvious to us that the signaling domains are exported to the periplasm. However, we understand this is not obvious for the readers. Following the reviewer’s suggestion we have included a sentence clarifying this point in the revised version.**

3. Page 11, lines 241 and 249. Where authors include a call to Fig. 5B, they probably mean Fig. 5A. In addition, in line 258, the call to Fig. 5C seems to correspond to Fig. 5B (there is no panel C)

**We apologize for this error. These panels have been now integrated as Figure 2C.**

4. I am not familiar with sedimentation velocity analytical ultracentrifugation, so that my comment might be wrong, but please note that the sedimentation coefficient values indicated in the text (page 11) for the different proteins analysed are always twice higher than those that I would (perhaps incorrectly) deduce from Fig. 5A. Please check the scale of the X-axis, or disregard this comment.

**The sedimentation coefficient values given in the text are the values obtained upon correction of the raw data shown in the Figure to the standard conditions. These standard conditions are 20 °C and water solvent (indicated with the suffix s20,w in the text), while the data shown in the figure correspond to the values obtained from the AUC experiment performed at 8 °C and in a buffer containing glycerol, which makes the density and viscosity of the solvent very different from the 20,w standard conditions. Correction of the raw data is performed through a simple correction factor that uses the density and viscosity of the solvent at both the experimental conditions, and at 20 °C and water. Consequently, the sedimentation coefficient numbers in the text and in the Figure differ notably. Correction of the raw data is a common practice in AUC to normalize different assays to be able to compare experiments performed in different conditions (i.e. different buffers or temperatures). We realize that this was not explained properly in the manuscript and apologize for this. We have clarified this issue in the Results and Materials and Methods sections, and the Figure legend of the revised version.**

**Reviewer #2:**

Summary:

Gram-negative bacteria utilize TonB-dependent outer-membrane receptors (TBDRs) to acquire exogenous nutrients from their environments. Among such cargo is iron, often in the form of iron chelates sequestered within siderophores. Many of these TBDRs play accessory roles in signal transduction, upregulating their own production in the presence of their substrate. These TonB-dependent transducers (TBDTs) contain an additional N-terminal signaling domain (SD) which participates in the pathway, ultimately resulting in release of a sigma factor which aids upregulation of transcription for the TBDT. While certain aspects of this pathway are understood, questions remain about the interactions between the TBDT and its cognate anti-sigma factor, as well as the specific role of the SD within the pathway. The current manuscript by Wettstadt et al builds on the current signaling model by demonstrating that in the absence of the inducing signal, the SD of one TBDT, FoxA, interacts with the cognate anti-sigma factor, FoxR, stabilizing it and preventing proteolytic degradation and subsequent sigma factor release. Using a two-hybrid binding assay, they identified specific interacting domains in the proteins. The authors used AlphaFold to predict the structure of the FoxA-SD/FoxR complex, and from this, identified candidate amino acid residues in the proteins which are important to the binding interface. The same two-hybrid system was used to validate key residues. Lastly, they show that the SD of FoxA is cleaved in a process involving CtpA and Prc, building off previous work from this group which described the role of Prc and CtpA in the signaling process.

The findings presented here present an important step in understanding signal transduction by TBDTs. However, the manuscript in its current form is not suitable for publication in PLOS Biology and the impact of this work on the field is not necessarily well suited to PLoS Biology. Several issues are outlined below, which the authors are invited to consider prior to resubmission.

1. Figure 6 and its associated results section cannot stand alone and be discussed as though it is experimental data. The model shown in this figure was generated using AlphaFold and is therefore a predicted model only, so the results from the prediction must be discussed and considered in this context. The information presented in this section can stay, but a suggestion is to revise the figure and use it to show a comparison between the current AlphaFold model and the already solved crystal structure of the PupB/PupR complex. In this depiction, the authors can highlight similarities between the solved PupB/PupR interface and the predicted FoxA/FoxR interface. This then naturally flows into the data from figure 7 in which important residues are directly tested and validated. Regardless of how the authors choose to address this, if the AlphaFold model is to remain in the manuscript, the supplementary information should include both a portrayal of the model colored according to B-factor, highlighting the relevant domain(s), as well as any relevant PAE matrices. The text sections as well should be modified to reflect that these are inferences from a predicted model, not "structural modeling data".

**We consider that the information obtained from the AlphaFold models has been discussed in the context of a prediction. We have explained properly how the models have been obtained and we always refer to them as structural models or structural predictions (rather than protein structure or crystal structure), which by definition implies that they are computer-generated structures. We have however slightly modified the text in this section to put more emphasis on this to avoid possible misunderstanding. Following the reviewer´s suggestion, we have included a new figure in the supplemental information (Fig. S7) with a structural alignment between the AlphaFold FoxR/FoxA model and the PupB/PupR complex, as well as the PAE matrix for the interaction. Because structural models do not contain a B-factor value, we have included the structural model colored by pLDDT instead. We refer to this Figure in the results section.**

2. Concerning the reworked figure 6, please modify the color scheme and label font/placement. The current version is difficult to interpret in its current from.

**We have modified the figure to make some of the labels easier to read.**

3. The results section concerning the AUC data presented in figure 5 is not interpretable in its current form. My combined PDF shows panels A and B for this figure, but the text does not refer to 5A at any point and refers to panel 5C which does not exist. It is not clear if this is simply a continuity/labeling error in the text because this section also refers to protein species with sedimentation coefficients which do not correspond to what is shown in the figure. For example, line 241 refers to FoxR-peri at 2.1 S, but this does not seem consistent with what is shown in the figures. This section should be revised for consistency and clarity.

**The panels showing the AUC data were named incorrectly in the text and we apologize for this error. These panels have been now integrated as Figure 2C. Regarding the discrepancy between the values given in the text and shown in the AUC Figure, please read our answer to the point 4 of reviewer 1 who has raised the same question. As stated there, this issue has been clarified in the revised version of the manuscript (see AUC results section, materials and methods and Fig 2C legend).**

4. For figure 5A, change the labels to reflect the actual protein domains used. FoxR-peri and FoxA-SD, not simply FoxR and FoxA.

**Labels have now been changed accordingly. The symbol of microM (μM) has also been changed as an error was detected.**

5. Figure 4C is out of place. The results section describing these data is a full 4 pages after the section describing 4A and B. Further, interpretation of 4C requires the reader to have already seen figure 6 in order to know why the STND domain and the relevant stretch of amino acids is important. This panel should be removed from figure 4, and in my opinion added to figure 7 instead.

**We agree with the reviewer and therefore this Figure has now been integrated with the structural model data as Figures 3A and 3B, respectively.**

6. Throughout the results section, be mindful of proper use of past tense to describe the results of the experiments. The blot showed that something happened, the assay demonstrated, etc. However, statements about interpreting the results ("these results suggest…") can and should be in present tense.

**This has been corrected.**

7. For figure 7A, move the "WT like" FoxR-C / FoxA-SD data point to either the top of the graph or the bottom with the other controls.

**The data is already placed with the other controls just after the positive and negative controls of the assay. We believe there is no need to move this data point to another location.**

8. Line 368 - I'm not sure I agree with the claim that a single protein band is seen in the PAO1 sample + ferrioxamine. It is faint, but the same lower MW band as is seen in the Δprc + ferrioxamine lane is visible. The overall conclusion that absence of Prc reduces CSS activity, presumably due to loss of the SD from FoxA as suggested by the western blot, remains valid.

**Please note that the band in the PAO1 sample + ferrioxamine is slightly lower than the band seen in the Δprc + ferrioxamine lane. We have changed the contrast of this figure to show better that these bands are not the same. Moreover, the PAO1 + ferrioxamine band is also detected in the negative controls (PAO1 – ferrioxamine and foxA-Tn mutant), indicating that it is not a product of the FoxA receptor. This can be also noted in the biological repetition of the blot shown in the manuscript (below).**

9. Loading controls should be shown for the blots in Figure 2B and 8

**Because we did not perform quantification of band intensities in these blots and only the presence or absence of the cleavage product is the important outcome, we consider that the use of Ponceau staining as loading control (as described in Materials and Methods) is sufficient in this case. Furthermore, detection of the non-cleaved protein band in these blots also serves as loading control.**
